# Wrist motion is distinct between touch screen and manual or digital devices

**DOI:** 10.1371/journal.pone.0290973

**Published:** 2023-10-09

**Authors:** Mandi J. Lopez, Catherine Takawira, Mary P. Fox, Pengju Wang, Evan Boatwright, Thomas Lucak, Chin-Chi Liu, Bryce Fugarino

**Affiliations:** 1 Department of Veterinary Clinical Sciences, School of Veterinary Medicine, Louisiana State University, Baton Rouge, LA, United States of America; 2 Department of Orthopaedic Surgery, School of Medicine, Louisiana State University Health Sciences Center New Orleans, New Orleans, LA, United States of America; Brunel University London, UNITED KINGDOM

## Abstract

**Background:**

Restricted motion during touch screen device use may contribute to wrist overuse injuries. Wrist radioulnar deviation and extension while using touch screen devices and digital or manual counterparts in male and female medical professional dominant and non-dominant hands were quantified to test the hypothesis that mobile touch screen device use reduces wrist motion.

**Methods:**

An active motion detection system was used to record wrist motion of 12 participants while: tablet swiping and turning book pages; raising a cell and traditional phone to the ear; texting and typing; and entering numbers on a cell phone and manual calculator. Medial and lateral wrist surface range of motion (ROM) and minimum and maximum wrist radial-ulnar deviation and flexion-extension were quantified.

**Results:**

Device, sex and handedness effects were determined (P<0.05). Maximum medial radial deviation and ROM were greater using a cell versus traditional phone. Maximum medial radial deviation was higher in the nondominant wrist during backward tablet swiping and while backward page turning versus tablet swiping. Maximum and minimum medial extension angles and ROM were greater while typing versus texting. Female nondominant hand maximum lateral extension and ROM were greater for typing versus texting and maximum medial extension and lateral extension ROM greater during manual versus cell phone calculator use with handedness combined. Maximum lateral extension and ROM were greater in females versus males using manual calculators.

**Conclusions:**

Sex and handedness should instruct touch screen, digital and manual device design and use for optimal performance and injury prevention.

## Introduction

Complex wrist anatomy and nearly continuous motion during daily activities contribute to high propensity for overuse injuries [1]. Repetitive motions within or beyond the normal joint range of motion (ROM) as well as prolonged static positioning contribute to musculoskeletal disorders [2–4]. Digital devices constitute a rapidly enlarging component of work and leisure activities, and frequent, extended use is associated with musculoskeletal injuries [5–14]. Quantification of wrist motion associated with device use is central to design, posture and positioning recommendations, and guidelines for safe and efficient use [7].

Wrist angles and ROM are typically measured in flexion-extension and radial-ulnar deviation as two- or three-dimensional angles, and dynamic wrist kinematic measures requires real-time data collection technology like optical motion capture systems [15]. The wrist joint axis is often defined as a point between the radial and ulnar styloid processes with the wrist joint center just distal to the midpoint of that axis [16–20]. Wrist joint angles and ROM are often indirectly derived from vectors between bony and virtual landmarks with the wrist center defined as a designated vertex, though joint coordinate systems and joint center triangulation vary [4, 15, 18, 19, 21–24]. Given complex carpal joint kinematics and distinct joint surface motion, direct measures of the medial and lateral joint surface maximum and minimum angles during touch screen, manual and digital device use may elucidate pathomechanics of wrist pain associated them [25].

The functional range of motion for the human wrist is determined during activities of daily living but do not usually include mobile or digital devices [26]. Device size, weight and actions required for use tend to decrease with advancing technology, potentially leading to less wrist excursion. Constrained motion and lack of a neutral resting position during is thought to contribute to musculoskeletal pain and injury [4, 13, 14]. Wrist motion can vary between males and females and dominant and nondominant limbs for a given task, so each should be considered for device use [23, 27–30], This study’s objective was quantification of the minimum and maximum, radioulnar deviation and flexion-extension of the lateral and medial wrist surfaces while using touch screen devices and their digital or manual counterparts in dominant and nondominant hands of male and female medical professionals to test the hypothesis that mobile touch screen device use reduces wrist motion.

## Materials and methods

### Participants

The study was approved by the institutional review board (Protocol #9078) and study subjects gave informed written consent to participate. Equal numbers of male or female medical school personnel with left or right dominant hands completed a series of activities in identical order. Inclusion criteria included: (1) 20 to 40 years old; (2) single dominant hand (no ambidexterity) according to the Edinburgh inventory [31]; (3) no history of upper extremity musculoskeletal pathology; (4) no current neurological deficits; (5) no joint pain or swelling. A licensed physician examined limbs prior to study participation. Data was obtained from 6 males (29.8 ± 1.8 years (mean ± standard error of the mean (SEM)) and 6 females (28.8 ± 2.1 years). Participant number was based on an a priori power analysis of mean difference in pre- and post-training scores for dominant and non-dominant hand computer mouse use [32].

### Devices

**Fig 1:**
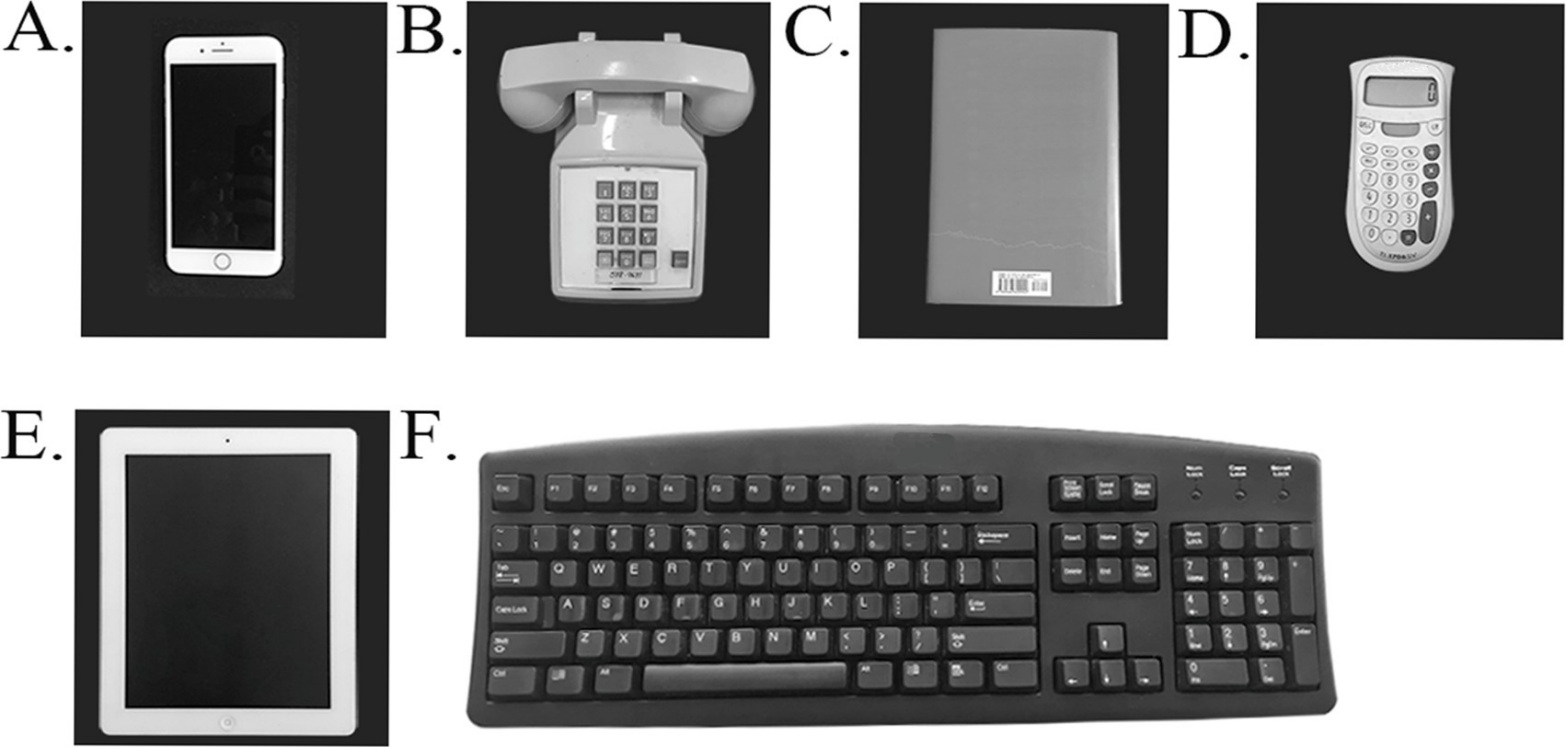
Device illustration. Photographs of devices tested in the study including a cell phone (A), traditional phone receiver (B), book (C), manual calculator (D), tablet (E), and keyboard (F).

Devices included in this study (Fig 1) included a cell phone with a touch screen (138×67×7 mm, diagonal length 119 mm, 148 g, Apple Inc, Cupertino, CA), traditional phone receiver (200×30×20 mm, 246 g, Cortelco Inc, Corinth, MS); book (234×155×25 mm, HarperCollins Publishers LLC, New York, NY); manual calculator (145×81×18 mm, 65 g, Texas Instruments Inc, Dallas, TX); tablet with a touch activated display screen (241×186×9 mm, diagonal length 246 mm, Apple Inc, Cupertino, CA); and computer keyboard (460×160×20 mm, incline angle 6°, Dell Inc., Round Rock, TX).

### Procedures

**Fig 2:**
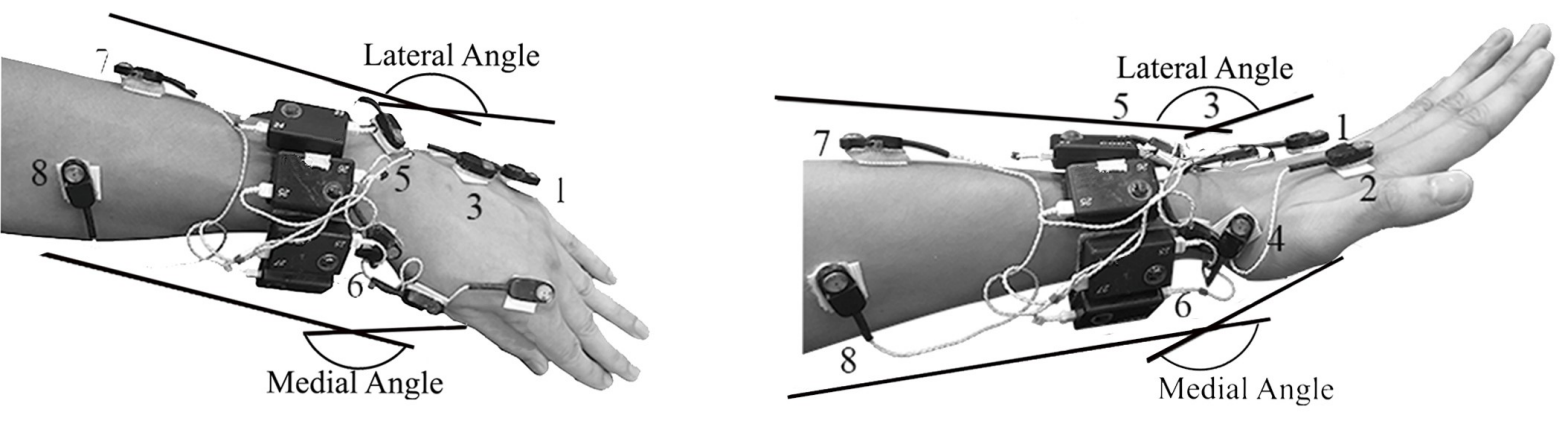
Marker Placement on a Left Hand with the Angles Shown for Deviation (Left) and Extension (Right). The carpal angle vertex on the lateral joint surface was formed by the intersection of vectors formed by markers 1 and 3 and markers 5 and 7. The carpal angle vertex on the medial joint surface was formed by the intersection of vectors between markers 2 and 4 and markers 6 and 8. For purposes of illustration, the lateral surface angle is elevated in the extension figure on the right.

A total of 8 wireless infrared markers (2 × 1.5 × 1 cm) of a 3D active motion detection system (Charnwood Dynamics Ltd) were affixed to the skin over anatomical landmarks on the medial and lateral surfaces of the forearm, wrist, and hand: 5th metacarpal head, hamate, ulnar styloid, ulna midpoint, 2nd metacarpal head, trapezium, radial styloid, and radius midpoint. The markers and two marker drive boxes were attached to the skin with pieces of VELCRO^®^ adhered to the skin with wig adhesive and to the markers and drive boxes with cyanoacrylate glue. The medial surface carpal angle vertex was the intersection of vectors between markers on the 2^nd^ metacarpal bone base and trapezium and markers on the radial styloid and radius midpoint. The lateral surface carpal angle vertex was formed by the intersection of vectors formed by markers on the 5^th^ metacarpal bone head and hamate and markers on the ulna styloid and ulnar midpoint (Fig 2).

### Activities

Participants were seated in an office chair at a desk that was adjusted in height so the elbow rested on the table while flexed at about 90° with the shoulder relaxed. Each performed 4 activities with the dominant hand first. Immediately prior to each activity, subjects practiced with the designated hand three times. A tone triggered participants to start three repetitions of an activity within 15 seconds when another tone sounded. During activities, participants rested their forearms on the desk surface, and between repetitions, rested their hands and forearms on the desk surface in a prone (neutral) position [33]. Activities included: raise a cell phone from the desk surface to the ear on the ipsilateral side as the hand and return it to the desk; raise a traditional phone receiver from the cradle to the ear on the ipsilateral side as the hand and return it to the cradle; swipe three times forward and then three times backward on a tablet on the desk surface; turn three pages forward and three pages backward in a book on the desk surface; text the sentence “The red fox jumped over the sleeping brown hound” holding a cell phone at a comfortable distance from the face using two hands; type the sentence on a computer keyboard using both hands; enter the numbers “10, 8, 6, 4, 2, 0” on a cell phone calculator with it on the desk surface; enter the numbers on a manual calculator on the desk surface. Two sensing units (CX1, Charnwood Dynamics Ltd) were situated 2 m from and facing the desk with the center of each approximately 20 cm above the desk surface. The origin of the virtual coordinate axis was on the desk surface at the center and oriented with the positive z, y, and x axes upward vertically, toward the desk front and toward the participant’s right, respectively.

### Data reduction procedures

**Fig 3:**
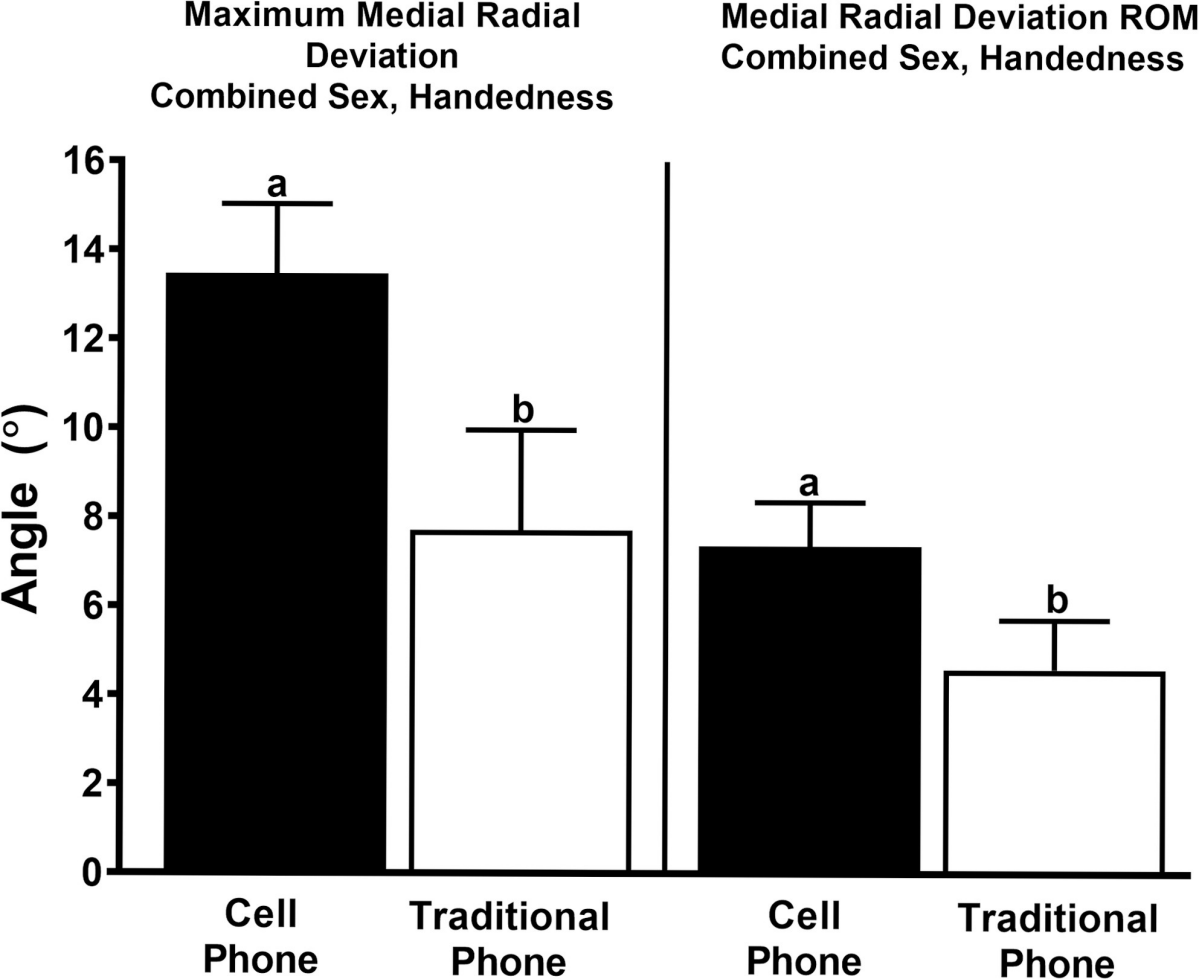
Standard versus cell phone wrist motion. Maximum medial radial deviation angle and range of motion (ROM) while raising a standard phone receiver or cell phone to the ear (least squares mean ± SEM). Columns with different letters are significantly between devices (p<0.05).

Data was collected at 200 Hz and included when marker visibility was 80–100% for a repetition. Angles were selected for each activity that were consistently measurable and corresponded to the predominant motion. Radial deviation was quantified for raising a cell phone or receiver to the ear and swiping or turning pages backward, ulnar deviation for swiping or turning book pages forward, and extension for texting, typing, and entering numbers on a cell phone and manual calculator. Two-dimensional, minimum and maximum extension (y-z plane) and radial and ulnar deviation (x-y plane) angles of the lateral and medial joint surfaces were recorded by the system software (Coda cx1, Charnwood Dynamics, Ltd.) and exported to spreadsheet software (Excel with Microsoft 365, Microsoft Corp.). The software setup included use of individual marker identities to create the medial and lateral free vectors, $\overset{↔}{31}$ and $\overset{↔}{57}$ and $\overset{↔}{68}$ and $\overset{↔}{42}$, respectively (Fig 3). The two vectors on each surface were used to define the medial and lateral vector angles in the system software in the x-y and y-z planes. Prior to each activity, a baseline angle was determined in the appropriate plane at the resting (prone) position. As indicated, the angles were recorded at 200 Hz during each repetition of all activities. The minimum and maximum angles used for statistical analysis were the absolute values of the difference between the baseline angle and the minimum and maximum angles recorded by the system for each repetition. The ROM was the absolute value of the difference between the calculated minimum and maximum angles except for swiping or turning book pages forward and backward. For those measures, the radioulnar ROM was the absolute difference between the calculated maximum ulnar and maximum radial deviation, the maximum angles in each direction recorded by the system, minus the baseline values. Marker locations permitted repeatable placement with which to generate the vectors used to define the measured angles. The angles were intended to represent direct measures like those measured on the lateral and medial joint surfaces with a manual goniometer.

### Statistical analysis

Results are presented as least squares mean (LSM) ± SEM. Normal data distribution was confirmed with the Shapiro-Wilk test. A mixed ANOVA model with fixed effects of sex, handedness, activity, and their interactions, and random effect of participant was used to evaluate differences between sexes, touch screen and digital or manual devices, and dominant versus nondominant hands for each wrist surface. Fixed effects were evaluated for each activity with Fisher’s least significant difference post-hoc comparisons. Results were combined for fixed effects that were not significant for an activity. All statistical analyses were performed with JMP^®^ Pro v. 14 and significance considered at *P* ≤ 0.05.

## Results

All results are provided as LSM ± SEM (Tables 1 and 2). The mean ± SD for each outcome is also included (Tables 3 and 4). Statistically significant differences are depicted in graphs (LSM ± SEM). The wrist surface, medial or lateral, and direction of angular deviation from a neutral position, radial, ulnar, flexion or extension, are indicated for each minimum and maximum angle. For ROM, the wrist surface and direction of angular deviation are included.

**Table 1:** Lateral and medial wrist surface radial or ulnar deviation and extension angles (least square mean +/- SEM) for distinct tasks.

Measure (°)	Sex^a^	Hand^b^	Cell Phone	Traditional Phone	Swiping Backward	Turning Book Page Backward	Measure (°)	Swiping Forward	Turning Book Page Forward	Measure (°)	Cell Phone Texting	Keyboard Typing	Entering Numbers Cell Phone Calculator	Entering Numbers Manual Calculator
**Maximum Lateral Radial Deviation**	F	D	9.1±1.9	5.6±2	40.0±7.6	39.4±8.4	**Maximum Lateral Ulnar Deviation**	30.8±5.6	31.9±10	**Maximum Lateral Extension**	23.7±3.9	17.1±4.1	23.7±7.0	28.4±6.1
		ND	6.7±1.7	7.5±2.0	40.9±6.9	39.8±8.2		33.3±4.2	38.0±6.1		5.9±4.7	29.1±3.8	15.4±5.0	31.8±6.0
	M	D	6.8±2.1	9.0±1.8	36.6±7.2	42.2±8.4		34.7±4.8	34.3±8.0		14.1±4.5	18±7.2	20.8±5	18.4±4.7
		ND	7.8±1.5	10.2±1.6	32.6±7.5	27.1±10.4		33.3±5.6	25.3±7.0		16.7±5.7	13.4±5.1	18.8±5.1	12.9±5.3
**Minimum Lateral Radial Deviation**	F	D	4.6±1.2	2.3±1.2	-	-	**Minimum Lateral Ulnar Deviation**	-	-	**Minimum Lateral Extension**	10.4±0.5	9.0±1.4	13.2±3.4	12.8±2.9
		ND	3.8±1.1	3.8±1.3	-	-		-	-		3.8±1.2	11.3±1.4	10.9±2.4	8.1±2.9
	M	D	2.2±1.4	3.6±1.2	-	-		-	-		3.2±1.8	6.3±4.5	10.5±2.4	8.2±2.2
		ND	3.3±0.9	5.0±1.0	-	-		-	-		2.6±3.3	5.7±2.5	4±2.5	5.6±2.6
**Maximum Medial Radial Deviation**	F	D	14.8±4.1	8.4±4.0	11.4±8.5	32.3±11.2	**Maximum Medial Ulnar Deviation**	25.1±9.2	61.2±19.2	**Maximum Medial Extension**	14.3±2.9	32.4±3.0	18.1±3.9	25.5±4.4
		ND	12.4±2.6	8.2±3.1	40.0±6.4	59.5±7.7		38.5±6.3	48.3±9.4		9.0±3.1	21.8±3.0	18.6±4.3	28.7±4.6
	M	D	15.2±2.5	8.7±3.0	15.1±7.8	28.3±9.8		21.5±10.2	35.9±9.3		7.8±3.4	16.9±3.5	16.6±5.1	16.1±4.7
		ND	11.5±2.6	5.6±6.5	38.7±9.0	33.3±8.6		36.1±12.4	35.8±10.7		12.2±4.1	23.9±4.1	27.1±5.5	26±4.6
**Minimum Medial Radial Deviation**	F	D	6.9±2.8	3.6±2.7	-	-	**Minimum Medial Ulnar Deviation**	-	-	**Minimum Medial Extension**	8.1±1.9	14.9±2.2	7.4±2.3	12.1±2.7
		ND	6.5±1.7	4.9±2.2	-	-		-	-		5.1±2.0	12.8±2.2	14.0±2.6	10.3±2.8
	M	D	7.1±1.7	5.2±2.1	-	-		-	-		3.5±2.1	7.7±2.2	8.8±3.1	5.9±2.8
		ND	5.0±1.8	1.1±5.2	-	-		-	-		2.6±2.5	6.0±5.2	8.8±3.4	7.7±2.7

^a^F: Female; ^a^M: male

^b^D: Dominant hand; ^b^ND: Nondominant hand

**Table 2:** Lateral and medial wrist surface range of motion range (least square mean +/- SEM) for distinct tasks.

Measure (°)	Sex	Hand	Cell Phone	Traditional Phone	Measure (°)	Cell Phone Texting	Keyboard Typing	Cell Phone Calculator Entering Numbers	Manual Calculator Entering Numbers	Measure (°)	Swiping Forward	Turning Book Page Forward	Swiping Backward	Turning Book Page Backward
**Lateral Radial Deviation ROM**	F	D	4.7±1.1	3.5±1.1	**Lateral Extension ROM**	14.9±6.6	9.9±2.2	10.0±6.1	17.6±5.1	**Lateral Radioulnar ROM**	13.1±6.8	19.6±20.9	21.5±10.4	15.6±10.9
		ND	3.1±0.9	3.8±1.1		4.4±2.4	18.7±3.4	5.5±4.1	24.4±5.1		17.6±3.8	7.8±5.7	24.0±5.8	18.9±7
	M	D	4.5±1.2	5.3±1.0		9.8±3.8	12.1±7.0	11.3±4.2	10.6±3.9		16.9±4.5	12.0±7.0	20.5±6.3	15.3±7
		ND	4.8±0.8	5.3±0.9		16.0±5.7	5.1±4.7	14.8±4.3	8.0±4.5		6.8±9.6	14.5±13.5	23.7±13	17.9±10
**Medial Radial Deviation ROM**	F	D	8.5±2.1	5.0±2.0	**Medial Extension ROM**	5.8±2.5	13.4±2.5	11.0±3.1	13.3±3.9	**Medial Radioulnar ROM**	11.2±5.4	25.6±10.3	14.9±15.5	17.6±12.5
		ND	5.8±1.4	3.2±1.6		4.3±2.6	13.8±2.5	4.9±3.7	18.4±4.2		12.3±3.9	11.1±4.8	19.7±7.8	28.3±10.3
	M	D	8.4±1.4	4.8±1.5		4.1±2.9	9.4±2.9	8.8±4.6	11.1±3.9		19.3±5.1	15.2±7.7	19.6±8.9	13.6±11.4
		ND	6.8±1.4	5.4±2.5		10.4±3.5	17.0±3.3	17.9±5.2	18.5±3.9		2.0±7.7	19.2±6.8	15.7+13.4	32.9±18.5

^a^F: Female; ^a^M: male

^b^D: Dominant hand; ^b^ND: Nondominant hand

**Table 3:** Lateral and medial wrist surface radial or ulnar deviation and extension angles (mean +/- SD) for distinct tasks.

Measure (°)	Sex	Hand	Cell Phone	Traditional Phone	Swiping Backward	Turning Book Page Backward	Measure (°)	Swiping Forward	Turning Book Page Forward	Measure (°)	Cell Phone Texting	Keyboard Typing	Entering Numbers Cell Phone Calculator	Entering Numbers Manual Calculator
**Maximum Lateral Radial Deviation**	F	D	9.3±4.4	5.9±43.0	33.8±8.6	32.7±20.3	**Maximum Lateral Ulnar Deviation**	31.0±11.8	33.1±10.7	**Maximum Lateral Extension**	24.0±9.5	19.8±7.7	23.1±11.9	31.2±16.7
		ND	8±5.0	7.3±7.0	39.8±21.3	33.7±11.1		33.4±15.3	39.5±17.8		5.6±1.5	29.8±14.3	17±5.6	32.9±17.7
	M	D	6.8±3.3	8.9±4.8	37.0±18.2	43.5±19.4		35.0±15.1	33.3±18.1		13.8±8.0	13.9±6.5	22.4±13.6	19.1±8.5
		ND	8.2±6.2	11.5±5.1	29.3±8.4	21.1±5.9		33.1±6.3	24.8±9.0		17.3±11.2	11.8±11.5	19.7±9.8	14±4.7
**Minimum Lateral Radial Deviation**	F	D	4.6±3.4	2.3±1.6	-	-	**Minimum Lateral Ulnar Deviation**	-	-	**Minimum Lateral Extension**	9.4±4.0	9.0±7.4	13.2±4.7	12.8±6.2
		ND	4.5±3.8	3.6±5.6	-	-		-	-		3.3±1.8	11.0±6.0	10.9±6.6	8.1±3.8
	M	D	2.1±1.4	3.6±4.0	-	-		-	-		3.1±2.7	6.5±1.1	10.6±6.3	7.6±6.8
		ND	2.8±2.2	5.1±4.2	-	-		-	-		2.8±1.6	5.4±1.7	4±4.7	5.6±4.0
**Maximum Medial Radial Deviation**	F	D	14.4±1.8	8.4±4.3	19.8±2.7	42.4±35.3	**Maximum Medial Ulnar Deviation**	21.8±11.4	60.8±19.2	**Maximum Medial Extension**	14.4±7.0	32.2±5.7	18.7±7.7	24.4±6.4
		ND	12.5±9.5	7.4±6.3	37.8±21.4	57.7±23.7		38.0±15.9	43.0±25.2		8.3±3.8	24.9±10.5	17.8±9.2	25.7±6.8
	M	D	15.1±8.6	8.6±3.7	18.1±5.0	29.0±9.6		21.8±9.6	33.3±19.1		7.2±1.2	16.8±13.0	18.6±7.7	17.6±8.3
		ND	12.0±4.5	7.7±2.1	37.2±21.4	57.7±23.7		38.0±20.0	31.3±12.8		13.5±7.5	28.2±na	24.7±14.9	26.2±20.0
**Minimum Medial Radial Deviation**	F	D	5.4±3.0	4.0±3.3	-	-	**Minimum Medial Ulnar Deviation**	-	-	**Minimum Medial Extension**	8.6±6.2	14.3±3.6	7.6±5.0	12.2±8.5
		ND	5.4±5.9	4.7±3.7	-	-		-	-		4.1±3.3	11.7±9.1	12.7±9.2	9.3±3.9
	M	D	7.1±4.7	5.3±3.1	-	-		-	-		3.1±0.8	7.4±4.9	9.5±6.5	6.3±2.9
		ND	4.5±1.3	1.1±1.3	-	-		-	-		3.1±2.8	6.9±na	7.3±10.5	7.6±4.4

^a^F: Female; ^a^M: male

^b^D: Dominant hand; ^b^ND: Nondominant hand

**Table 4:** Lateral and medial wrist surface range of motion range (mean +/- SD) for distinct tasks.

Measure (°)	Sex	Hand	Cell Phone	Traditional Phone	Measure (°)	Cell Phone Texting	Keyboard Typing	Cell Phone Calculator Entering Numbers	Manual Calculator Entering Numbers	Measure (°)	Swiping Forward	Turning Book Page Forward	Swiping Backward	Turning Book Page Backward
**Lateral Radial Deviation ROM**	F	D	4.7±3.3	3.5±2.5	**Lateral Extension ROM**	14.6±12.8	10.2±9.3	9.9±11.4	18.4±15.5	**Lateral Radioulnar ROM**	18.6±18.9	30.0±12.8	23.8±14.7	28.1±19.2
		ND	3.1±2.0	3.8±2.1		2.3±2.2	19.2±13.6	6.1±3.8	24.8±17.4		25.9±18.7	31.4±19.4	31.6±25.0	23.0±11.7
	M	D	4.8±2.3	5.1±1.6		10.7±9.9	7.4±5.4	11.8±10.4	10.9±4.4		26.6±17.4	22.8±17.3	26.5±20.5	22.7±20.7
		ND	5.3±4.4	5.4±4.3		14.5±12.4	6.2±9.4	12.4±7.2	8.4±7.5		27.8±8.5	18.2±11.7	22.6±13.5	12.3±10.1
**Medial Radial Deviation ROM**	F	D	7.5±1.5	4.4±1.4	**Medial Extension ROM**	5.8±4.0	15.1±6.5	11.1±5.4	13.3±12.7	**Medial Radioulnar ROM**	12.3±10.4	42.05±na	14.6±3.7	32.9±25.5
		ND	5.9±3.5	3.8±4.0		4.3±3.2	12.5±9.3	5.1±2.6	18.4±7.7		26.2±17.7	36.4±26.1	28.7±21.1	26.3±26.5
	M	D	8±5.0	3.2±2.2		4.1±1.0	9.4±14.4	9±2.7	11.3±7.0		16.0±12.0	25.3±14.0	10.6±5.5	23.1±10.9
		ND	7.0±4.3	4.7±4.9		10.4±10.0	21.3±3.4	17.4±5.3	18.6±21.3		27.2±20.5	13.4±15.3	28.0±20.5	20.6±10.3

^a^F: Female; ^a^M: male

^b^D: Dominant hand; ^b^ND: Nondominant hand

**Fig 4:**
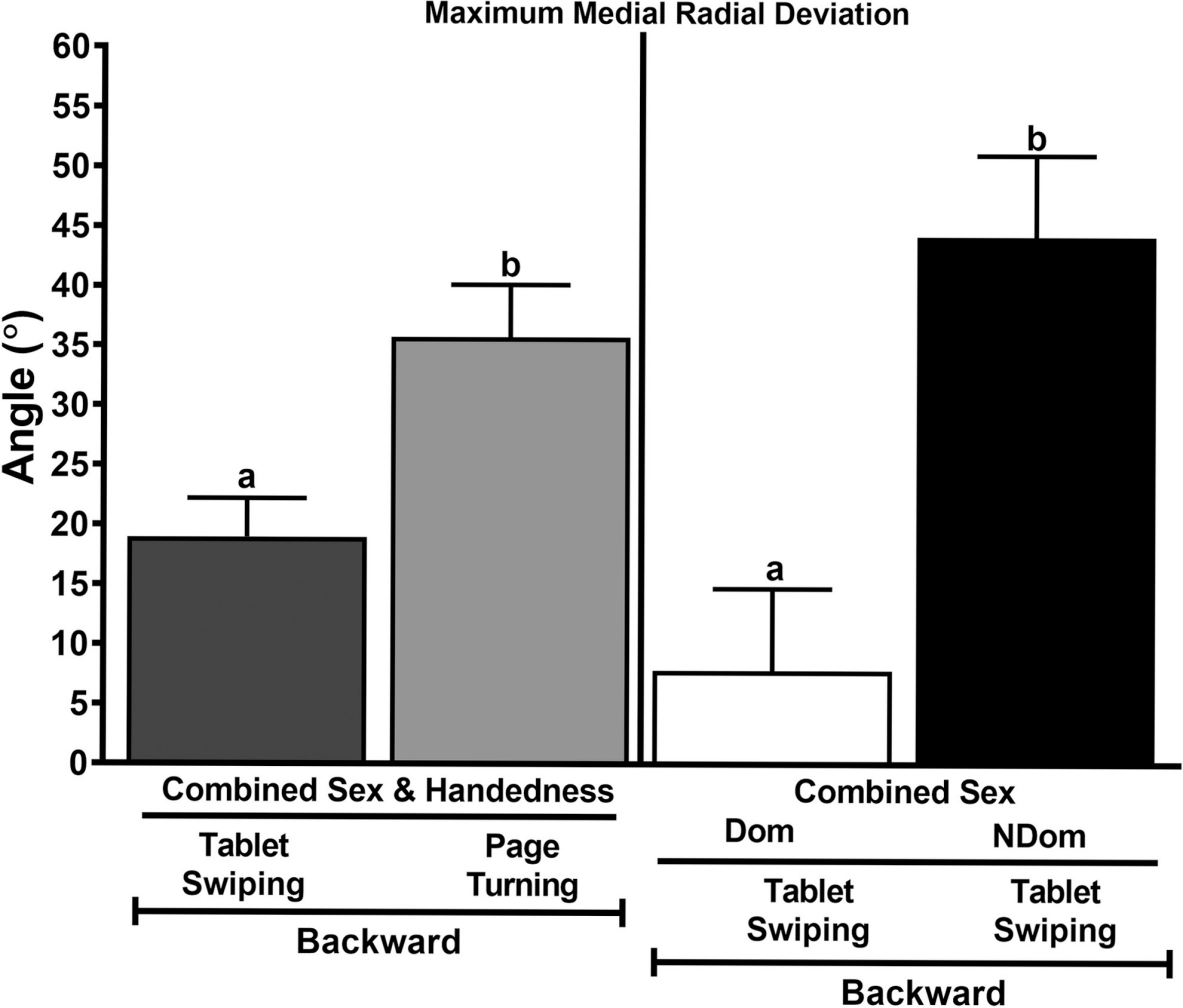
Page turning versus tablet swiping wrist motion. Maximum medial radial deviation (least squares mean ± SEM) while swiping on a tablet or turning book pages backward. Columns with different letters within comparison are significantly different (p<0.05).

**Fig 5:**
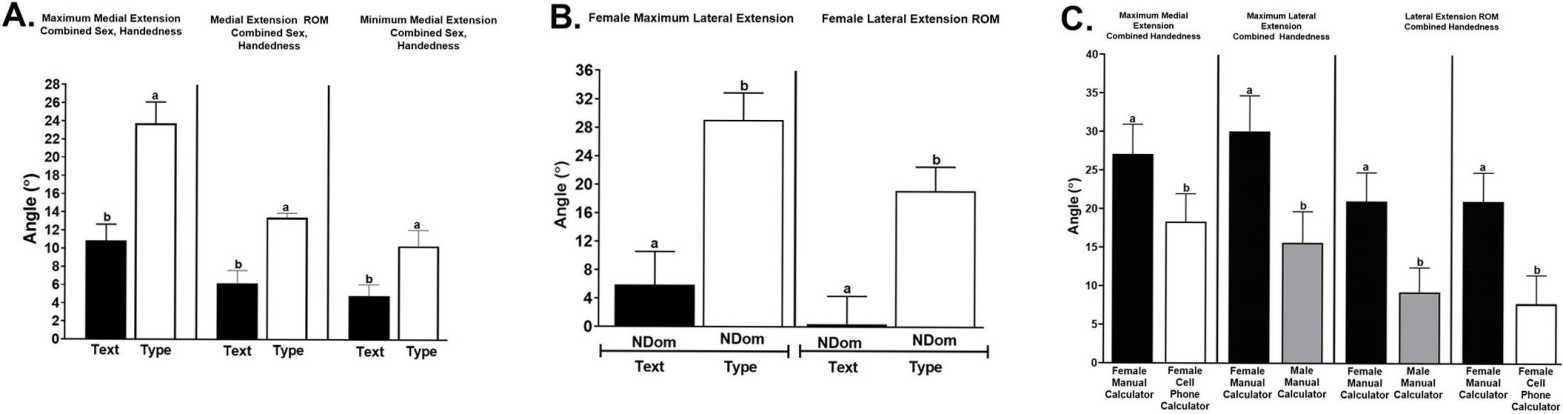
Typing versus texting and manual versus cell phone calculator wrist motion. Maximum medial extension and range of motion (ROM) and minimum medial extension (A) and female maximum lateral extension and ROM (B) while texting or typing, and maximum medial extension, maximum lateral extension, and lateral extension ROM while using a cell phone or manual calculator (C, least squares mean ± SEM). Columns with different letters within each comparison are significantly different (p<0.05).

The maximum medial radial deviation angle and medial radial deviation ROM were greater using a cell versus a traditional phone receiver (13.5 ± 1.6 versus 7.7 ± 2.3, *P* = 0.04; and 7.4 ± 1.0 versus 4.6 ± 1.12, *P* = 0.01) with sex and handedness combined phone (Fig 3). The maximum medial radial deviation angle was greater while turning pages versus swiping backward with sexes and handedness combined (35.7 ± 4.4 versus 19.0 ±3.2, *P* = 0.04), and the angle was greater in the nondominant versus dominant wrist with sexes combined (44.2 ± 6.8 versus 7.8 ± 6.9, *P* = 0.0024; Fig 4). With sex and handedness combined, the maximum medial extension angle and range of motion were greater while typing versus texting (23.8 ± 2.3 versus 10.8 ± 1.8, *P* = 0.0001; and 13.4 ± 0.5 versus 6.2 ± 1.4, *P* = 0.0001) and the minimum medial extension angle was smaller for texting (4.8 ± 1.2 versus 10.3 ± 1.7, P = 0.002; Fig 5A). The maximum lateral extension angle and ROM were greater while typing versus texting in the female nondominant wrist (29.1 ± 3.8 versus 5.9 ± 4.7, *P =* 0.0005; and 19.2 ± 3.4 versus 0.5 ± 3.9, *P* = 0.005; Fig 5B). With handedness combined, the maximum medial extension angle was greater while using a manual versus cell phone calculator in females (27.1 ± 3.9 versus 18.3 ± 3.7, *P =* 0.005), the maximum lateral extension angle was greater in females versus males while using a manual calculator (30.1 ± 4.6 versus 15.6 ± 4.1, *P =* 0.04), and the lateral carpal extension ROM was greater in females versus males while using a manual calculator (21.0 ± 3.7 versus 9.3 ± 3.2, *P =* 0.02) and in females while using a manual versus cell phone calculator (21.0 ± 3.7 versus 7.8 ± 3.7, *P =* 0.01; Fig 5C).

## Discussion

The hypothesis, mobile touch screen device use reduces wrist motion, was rejected because it was not true in all tested devices. Specifically, medial radial deviation and ROM were higher using a cell versus traditional phone. However, swiping on a tablet, texting, and using a cell phone calculator did appear to reduce wrist motion relative to manual or digital counterparts. Differences in wrist motion for specific devices were also evident between sexes and dominant versus nondominant hands, and many were distinct between medial or lateral wrist surfaces. The results indicate that wrist motion using touch screen and manual or key activated digital devices varies with joint surface and are affected by sex and handedness, important considerations for device design and use recommendations to prevent overuse injury.

Many detectable differences in wrist motion among devices in this study were with sex and handedness combined, presumably because their effects are inseparable for many activities. The higher medial radial deviation associated with cell phone use exemplifies this point. Cell phones tend to be wider than standard phone receivers, and the distance between the hand and ear with a cell phone is shorter than with a receiver. Wrist radioulnar deviation during merchandise bagging was greater when grasping 10 cm versus 5 cm diameter objects [34]. In this study, the greater radial deviation for cell phone use may be attributable to the larger grasp required, about 2 times that of the receiver, as well as closer proximity of the hand to the ear. Speakers that obviate the need for static hand positioning may reduce potential for wrist injury during frequent or prolonged verbal phone communication.

The maximum medial radial deviation for page turning versus swiping and maximum medial extension and ROM and minimum medial extension for typing versus texting were sufficiently distinct between devices, but not hand or sex, to be detected. However, motion was lower for touch screen devices. While the initial direction, radial or ulnar deviation, for backward page turning or swiping was different depending on the hand, left or right, in use, only medial radial deviation was significantly higher for page turning. The process of semi-grasping, elevating, and releasing a book page requires more complex wrist motion than swiping on a tablet, and swiping requires sustained contact in a more constrained space [4]. Greater medial extension angle and ROM for typing versus texting and maximum medial extension angle and lateral extension ROM during female manual versus cell phone calculator use could also be associated with device attributes, specifically key activation [35, 36]. Key activation required to text on a cell phone with a keypad requires greater thumb flexion-extension and ROM compared to a touchscreen [35]. As such, the act of key activation versus touch may increase wrist motion, though the effect would likely vary with key and keyboard design.

There is no universally accepted model of carpal bone motion, but current understanding is that the distal row, hamate, capitate, trapezoid, and trapezium, moves more uniformly than the proximal row, triquetrum, lunate, and scaphoid [25, 37]. Variable bony kinematics and kinetics may allow for unique medial and lateral joint surface motion. Wrist pain is often specific to the ulnar (lateral) or radial (medial) side from conditions like trapeziometacarpal joint osteoarthritis that preferentially impacts one sex [38–40]. The novel methods employed in this study went farther than measures taken at the forearm and hand midpoints and resulted in uniquely granular data to prevent, treat, and rehabilitate wrist musculoskeletal disorders resulting from touch screen devices and their manual and digital counterparts [3, 4, 19, 22–24, 35].

The angles and ROM measured in this study are not all extremes of wrist motion [19, 41]. Constrained motion and non-neutral position contribute to device-associated musculoskeletal disorders [4, 14, 42]. Repetitive, constrained wrist motion can result in increased upper limb antagonist muscle tension that contributes to reduced range of motion [33, 43]. The lower minimum medial extension angle and maximum medial extension and ROM for texting versus typing, and the lower maximum medial extension and lateral extension ROM for cell phone versus manual calculator use are consistent with constrained motion. Minimum angles provide information about constrained motion wrist motion beyond that captured by differences among maximum angles and may further understanding of wrist pain and injury.

Differences in wrist deviation and flexion-extension vary between sexes and hands [29]. Better dominant hand manual dexterity and greater nondominant hand non-instructive motion are typical, and dominant hand accuracy becomes more evident with task precision requirements [27, 28, 30, 44, 45]. Broadly speaking, sex and hand differences became evident during tasks in this investigation that required the most accuracy, including manual calculator use. Differences between sexes in maximum extension and ROM during the activity were both larger than reported differences in wrist flexion-extension ROM of large and small hands during piano playing [46]. Distinct radiocapitate kinematics in the female wrist are currently thought to be the reason for sex differences [41, 47, 48]. Smaller hand size can result in larger wrist ROM for an activity, and female hands are typically smaller than male [46, 49]. Differences between male and female dominant and nondominant hand accuracy and strength, including during digital device use have been confirmed [29, 47, 50]. Additionally, it is likely that personal experience like sports, instrument use, and strenuous manual tasks influence device use. Individuals with left-dominant hands often use their right hand for hand-specific devices and therefore may have more experience using their nondominant hand. Specific differences between dominant and nondominant hand use among sexes is beyond the scope of this study; however, the findings indicate important differences for sex and hand-specific device customization [28].

## Limitations

Results should be limited to the comparisons here within study limitations. Markers were on bony landmarks where subcutaneous tissue was minimal, so skin motion was minimized but not obviated. The markers and marker boxes attached to the forearm could have affected normal wrist motion of participants, and the effects might have varied among individuals. Task order was not randomized, though 10–15 minutes between tasks should minimize carryover effects. Due to busy medical trainee schedules, it was not possible to repeat tasks on different days to test repeatability. The small participant population, medical trainees in an age range with high wrist ROM, though consistent with comparable studies, may limit generalizability of study findings [24, 32, 41, 47, 51]. Pronation and supination that impact 3D angle measures were not included in the 2D angle measures [29].

### Conclusions

New information from this study that examined medial and lateral wrist motion during use of touch screen devices and digital and manual counterparts confirms distinct wrist motion between dominant and non-dominant hands that varies between male and female users. Maximum and minimum joint angles provide added information that may expand understanding of musculoskeletal disorders associated with the devices evaluated. Methods and results specific to wrist surface and maximum and minimum motion may also provide a platform for future studies. Taken together, results confirm that sex and handedness should guide recommendations for digital and manual device design and use to optimize performance and reduce injury.
